# Evaluation of community-based systems for the surveillance of day three-positive *Plasmodium falciparum* cases in Western Cambodia

**DOI:** 10.1186/1475-2875-13-282

**Published:** 2014-07-22

**Authors:** Jonathan Cox, Lek Dy Soley, Tol Bunkea, Siv Sovannaroth, Kheang Soy Ty, Song Ngak, Steven Bjorge, Pascal Ringwald, Steven Mellor, David Sintasath, Sylvia Meek

**Affiliations:** 1 Faculty of Infectious and Tropical Diseases, London School of Hygiene and Tropical Medicine, London, UK; 2 Malaria Consortium, Phnom Penh, Cambodia; 3 National Centre for Parasitology, Entomology and Malaria Control, Phnom Penh, Cambodia; 4 National Institute of Public Health, Phnom Penh, Cambodia; 5 University Research Co., Phnom Penh, Cambodia; 6 FHI 360, Phnom Penh, Cambodia; 7 World Health Organization, Phnom Penh, Cambodia; 8 World Health Organization, Geneva, Switzerland; 9 Malaria Consortium, London, UK; 10 Malaria Consortium, Bangkok, Thailand

**Keywords:** Malaria, Cambodia, Surveillance, Anti-malarials, Artemisinin, Case management, Community health worker, Village malaria worker, Operational research

## Abstract

**Background:**

Delayed clearance of *Plasmodium falciparum* parasites is used as an operational indicator of potential artemisinin resistance. Effective community-based systems to detect *P. falciparum* cases remaining positive 72 hours after initiating treatment would be valuable for guiding case follow-up in areas of known resistance risk and for detecting areas of emerging resistance.

**Methods:**

Systems incorporating existing networks of village malaria workers (VMWs) to monitor day three-positive *P. falciparum* cases were piloted in three provinces in western Cambodia. Quantitative and qualitative data were used to evaluate the wider feasibility and sustainability of community-based surveillance of day three-positive *P. falciparum* cases.

**Results:**

Of 294 day-3 blood slides obtained across all sites (from 297 day-0 positives), 63 were positive for *P. falciparum,* an overall day-3 positivity rate of 21%. There were significant variations in the systems implemented by different partners. Full engagement of VMWs and health centre staff is critical. VMWs are responsible for a range of individual tasks including preparing blood slides on day-0, completing forms, administering directly observed therapy (DOT) on days 0–2, obtaining follow-up slides on day-3 and transporting slides and paperwork to their supervising health centre. When suitably motivated, unsalaried VMWs are willing and able to produce good quality blood smears and achieve very high rates of DOT and day-3 follow-up.

**Conclusions:**

Community-based surveillance of day-3 *P. falciparum* cases is feasible, but highly intensive, and as such needs strong and continuous support, particularly supervision and training. The purpose and role of community-based day-3 surveillance should be assessed in the light of resource requirements; scaling-up would need to be systematic and targeted, based on clearly defined epidemiological criteria. To be truly comprehensive, the system would need to be extended beyond VMWs to other public and private health providers.

## Background

The Thai-Cambodian border has long been considered to be an epicentre of emerging resistance of *Plasmodium falciparum* to anti-malarial drugs, including chloroquine, sulphadoxine-pyrimethamine and mefloquine [1]. Artemisinin-based combination therapy (ACT) was implemented as the first-line treatment for uncomplicated falciparum malaria in Cambodia in 2000. Artemisinin resistance, expressed as reduced parasite clearance rates *in vivo*, was first detected in Pailin province in 2007–2008 [2] and has recently been reported in neighbouring Pursat province [3]. In late 2008 Cambodia and Thailand embarked on a joint strategy for the containment of artemisinin-tolerant malaria parasites in Southeast Asia (ARCE), which involved the introduction or scaling up of a number of interventions in key districts on both sides of the Thai-Cambodia border [4]. Within this strategy the presence of *P. falciparum* parasitaemia 72 hours after the initiation of treatment with an ACT (i.e*.* at day-3) has been used as a standard *in vivo* indicator of the possible presence of drug resistance [5]. In areas considered to be at high risk of developing artemisinin resistance (and particularly areas bordering zones where resistance has already been detected), there is a recognized need to develop surveillance systems through which health care providers can identify and report delayed clearance at day-3, both as a means of monitoring geographical patterns of resistance and as a basis for facilitating appropriate response activities [4].

Within Cambodia the ARCE strategy has supported the National Centre for Parasitology, Entomology and Malaria Control (CNM) in developing new approaches to malaria surveillance and stratification. This has included the introduction of a new system of routine case reporting that supports incidence-based stratification of malaria at the village level. It has also involved testing systems for real-time, community-level reporting of cases at the point of care using village malaria workers (VMWs). Since its introduction in 2001, the Cambodia VMW network has been substantially scaled up [6,7] and today covers a total of 1,576 villages in 18 malaria-endemic provinces (unpublished CNM data). VMWs are primarily tasked with performing rapid diagnostic tests (RDTs) on individuals suspected of having malaria and with treating or referring test-positive cases according to defined guidelines. They are also encouraged to conduct active case detection, follow up malaria patients and disseminate information on malaria preventive measures.

Between July 2010 and September 2011 a new system using community-based VMWs to detect, treat and report day three-positive cases of *P. falciparum* was piloted in selected districts of western Cambodia by CNM with technical support from University Research Co. (URC), FHI 360 and the Malaria Consortium. The primary objectives of the pilot phase were: (a) to assess the feasibility and viability of community-based systems to capture and disseminate information on day three-positive *P. falciparum* cases; and, (b) to collect and analyse basic epidemiological data relating to day three-positive *P. falciparum* cases. A secondary objective was to test the viability of a SMS alert system for providing CNM and other key stakeholders with real-time notifications of day three-positive *P. falciparum* cases. This paper focuses on the effectiveness of surveillance activities introduced at the community level through the VMW system.

## Methods

### The pilot system

Community-based surveillance systems were implemented at selected sites in Pailin, Battambang and Pursat provinces in western Cambodia. In each province, the pilot project was implemented by a different project partner (CNM in Pursat, URC in Battambang and FHI 360 in Pailin). Overall, the pilot system covered a total of 76 villages, all of which fell within ARCE containment zone 1, an area targeted for *P. falciparum* malaria elimination. Pilot surveillance activities started between July and October 2010, depending on the implementing partner, and were originally scheduled to run for six months. Delays in the start up of some activities, together with a smaller than expected number of malaria cases at some sites in the early months of the project, led to the pilot period subsequently being extended to run to the end of September 2011.

A common framework for pilot activities was agreed in advance by the three implementing partners and as far as possible aimed to integrate day-3 surveillance tasks within routine VMW activities, for example by carrying out refresher training, supervision and resupply of consumables during VMW monthly meetings. Within this common framework patients presenting to VMWs with suspected malaria were tested using an RDT, as per the standard VMW system. VMWs were then required to prepare thick and thin blood films for individuals testing positive for *P. falciparum* or mixed infections and to fill out relevant sections of a project-specific case report form. Blood slides were air-dried and stored for subsequent staining and examination by laboratory staff at the relevant supervising health centre. VMWs were required to administer directly observed therapy (DOT) for all *P. falciparum*-positive individuals using a standard three-day treatment course of Duo-cotecxin (dihydroartemisinin-piperaquine). In addition, VMWs were asked to prepare a follow-up blood slide for all individuals on day-3. After examining these slides, laboratory staff at the supervising health centre were responsible for completing the case report form and, in instances where the day-3 slide was positive for *P. falciparum*, to send a pre-coded SMS message to CNM using a dedicated phone number. Once the SMS was received by the CNM server, text alerts with details of individual day three-positive cases were relayed to relevant partners, including staff at district and provincial health departments.

Within this basic framework a certain amount of flexibility was retained to allow individual implementing partners to adjust their protocols to best suit the existing situation on the ground. For example, details concerning the logistics of patient follow-up (DOT and day-3 slide), the method of transporting blood slides from VMWs to health centres, local staffing and supervision arrangements, and staff remuneration were determined individually by partners. An important element of the evaluation was an assessment of the relative importance and merit of these variations within the overarching protocol.

### The evaluation

Qualitative data relating to system performance and provider experiences were collected through a series of semi-structured, open-response interviews with key informants. Separate interview guides for VMWs and health facility staff were prepared in advance on the basis of pre-evaluation field visits. Interviews were conducted with a cross-section of VMWs (n = 32) in 27 villages. Because only a small minority of villages had reported *P. falciparum* cases during the pilot phase, the selection of VMWs was essentially non-random but was stratified in such a way as to capture variations in village population size, malaria case loads and accessibility. Six supervising health facilities were also visited and all staff officially associated with the day-3 pilots (including health centre chiefs, clinical staff and laboratory staff) were interviewed. In addition, field staff from FHI 360 and URC were interviewed at provincial level and principal investigators were interviewed at the national level.

As part of this evaluation, quantitative surveillance data from the community-based pilot were collated and analysed. Implementing partners were asked to provide a range of data, including information on RDT and blood slide results and ‘process’ data relating to the timings of blood slide preparation, transport and examination.

This evaluation was led by the Cambodia Ministry of Health and all activities, including interviews, were conducted with the full participation of, and were supervised by, senior staff from CNM. In line with standard CNM practice it was required that participants provide informed verbal consent prior to interviews. The evaluation was approved by the Ministry of Health of Cambodia and constituted a core programmatic activity of CNM.

## Results

### Malaria case data

Table 1 presents summary data on day-0 and day-3 *P. falciparum* cases reported by VMWs during the pilot period. Across all sites a total of 297 day-0 blood slides were positive for *P. falciparum*, of which 28 represented mixed infections with *P. vivax*. At day-3, 63 out of 294 blood slides examined were positive for *P. falciparum* (additionally, two were positive for *P. vivax*), representing an overall day-3 *P. falciparum* positivity rate of 21.4%. The majority of day-3 *P. falciparum* cases (54/63; 86%) were detected at Ta Sanh in Battambang province, where the overall day-3 *P. falciparum-*positivity rate was 22%. In Pailin, the day-3 positivity rate, based on a relatively small denominator, was slightly higher, at 33.3% (7/21). At Pramaoy, Pursat, of the 25 day-3 slides examined, only two *P. falciparum* infections were detected at day-3 (positivity rate = 8%). No day-3 *P. falciparum* cases were detected at Trang (Battambang). In terms of basic demographic characteristics, 76% of day-0 *P. falciparum*-infected individuals were male and 78% of *P. falciparum-*infected individuals were aged 15 or over (corresponding figures for day-3 *P. falciparum* cases were similar, at 70 and 79%, respectively).

**Table 1:** Site-specific summary data for day-0 and day-3 cases generated by URC, FHI 360 and CNM community pilots

	***URC***		***FHI 360***	***CNM***	
	**Ta Sanh**	**Trang**	**Pailin**	**Pramaoy**	***Total***
Villages included in pilot	13	15	28	20	*76*
Villages reporting Pf cases	11	2	14	8	*35*
Number of day-0 slides prepared	276	6	60	27	*369*
Number of day-0 slides *P. f* positive	245	4	21	27	*297*
Number of day-3 slides prepared	245	3	21	25	*294*
Number of day-3 slides *P. f* positive	54	0	7	2	*63*
Overall % day-3+	22.0	0.0	33.3	8.0	*21.4*

Overall, fewer than half (35/76) of the villages included in the pilot studies reported any *P. falciparum* cases over the period of the project. This proportion varied substantially between sites; in Ta Sanh 85% of villages reported at least one *P. falciparum* case, while in Pailin, Pramaoy and Trang this figure was substantially lower (at 50, 40 and 13%, respectively).

**Figure 1:**
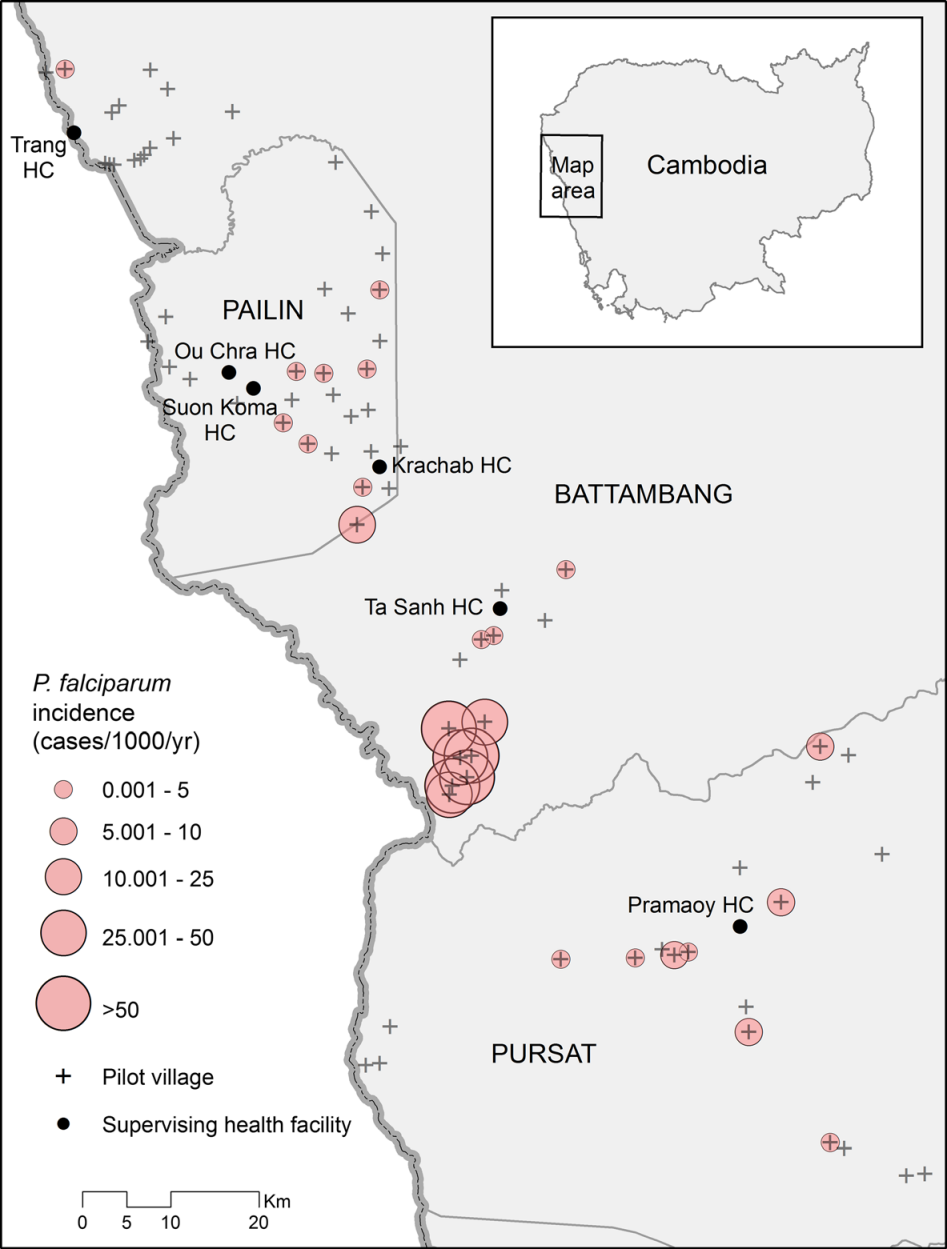
**Variations in village-level incidence of *Plasmodium falciparum *** **across pilot study sites in Battambang, Pailin and Pursat provinces, Cambodia.**

**Figure 2:**
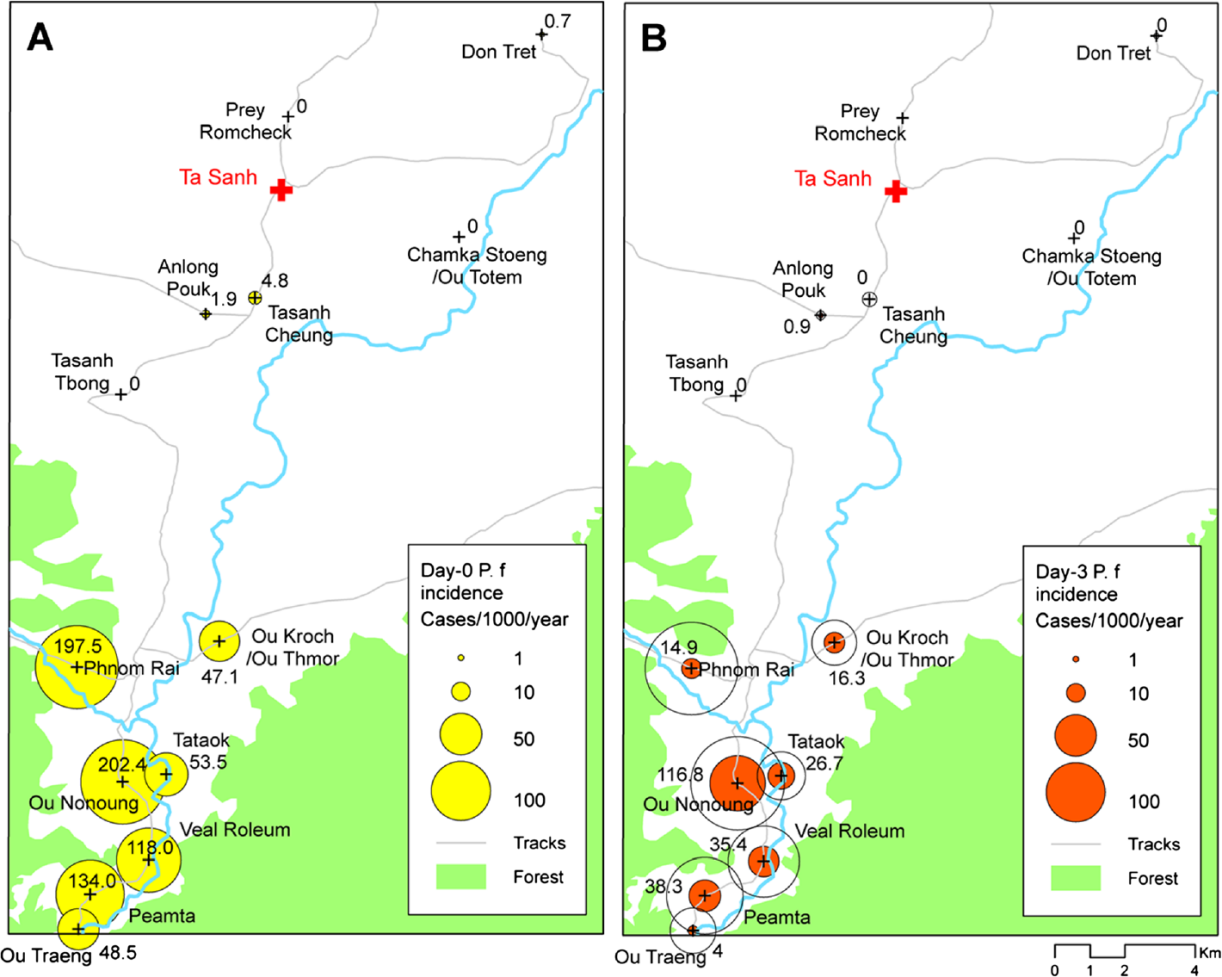
**Local spatial patterns of incidence of *Plasmodium falciparum *** **infection at day-0 (Panel A) and day-3 (Panel B) in the catchment of Ta Sanh health centre, Battambang province.**

Based on VMW data and using village population data collected through the ARCE containment project, spatial patterns of malaria incidence demonstrated marked heterogeneity across the study area (Figure 1), with the majority of day-0 cases (245/297; 82%) being reported by VMWs at Ta Sanh. This spatial heterogeneity was also evident within individual study sites for both day-0 and day-3 infections. To illustrate this point, Figure 2 shows local spatial variations in day-0 incidence rates (Panel A) and day-3 incidence rates (Panel B) among pilot villages within the Ta Sanh site. Most of the villages in the northern part of the catchment reported few, if any, day-0 *P. falciparum* cases. Malaria incidence was highest in villages at the southern end of the catchment, located closest to primary forest. In two villages (Phnom Rai and Ou Nonoung) day-0 *P. falciparum* incidence was around 200 cases per 1,000 population per year. However, day-0 and day-3 incidence rates were not strongly correlated. In the case of Ou Nonoung, for example, the day-3 positivity rate was 58%, which translated into a day-3 incidence rate of 116.8 cases per 1,000 per year. In contrast, the day-3 positivity rate in Phnom Rai was only 7.5%, which translated into a much lower rate of day-3 incidence of 14.9 cases per 1,000 per year (Figure 2).

Data from the pilot studies demonstrated temporal as well as spatial clustering, particularly in terms of day-3 positivity rates. Most notably, 26 of the 54 day-3 cases reported in Ta Sanh were detected in a four-week period between 26 September and 21 October. This translates into 48% of the day-3 *P. falciparum* cases being reported within a time-window representing 8% of the study period (by comparison 22% of day-0 *P. falciparum* cases were reported during the same period).

### Process indicators

In order to assess operational feasibility of the systems, data on the implementation were documented. When compiling datasets for the community-based pilot, implementing partners were asked to collate (where possible) additional information relating to the timing of various activities within the case reporting/management process, including day-0 and day-3 slide preparation, slide examination and the administration of DOT. In the event not all of these data were available from all partners. Notably, no data relating to the timing of slide examination or treatment were available for the CNM pilot at Pramaoy.

Table 2 summarizes process indicators relating to management of day-0 and day-3 slides that are common to both the URC and FHI 360 datasets. URC data from Trang are not included because of the very small sample size and a relatively large number of unresolvable errors among the dates entered. A notable feature of the data presented for Pailin is the relatively low rate of follow-up achieved at day-3; among 60 cases identified on day-0, 39 cases were referred to ongoing drug efficacy studies in Pailin (using different drug regimens) and were therefore not available for follow-up. Within the FHI 360 dataset process, information was only available for the 21 individuals who were followed up to day-3. All appeared to have received treatment on day-0 and all had their follow-up day-3 slide prepared on the correct day. Within the FHI 360 system day-0 and day-3 slides were transported individually by VMWs to the health centre to be stained and examined. Taking data for day-0 and day-3 slides together, 88% of slides were received by the health centre on the day they were prepared. The remaining 12% were sent the next day. The majority (83%) of day-0 and day-3 slides appear to be have been examined on the day they were received at the health centre, again, with the remainder being read the following day.

**Table 2:** Summary of key process indicators at FHI 360 and URC pilot sites

***Process indicator***		***Pailin (FHI 360)***	***Ta Sanh (URC)***
Number of day-0 slides prepared:		60	276
Number of day-3 slides prepared:		21	276
Treatment initiated:	*Same day (day-0)*	21	268
	*+1 day*		6
day-3 slide prepared:	*On day-3*	21	266
	*On day-4*	-	6
Blood slides* received by health centre:	*Same day*	37	242
	*+1 day*	5	164
	*+2 days*	-	30
	*+ 3 days or more*	-	14
Blood slides* examined at health centre:	*Same day as received*	35	216
	*+1 day*	7	184
	*+2 days*	-	44
	*+ 3 days or more*	-	34

*Aggregate of both day-0 and day-3 slides. Note for FHI information on dates of slide transport and examination are only available for 21 cases who were successfully followed up on day-3.

Process data for Ta Sanh were similarly impressive. The data on treatment delay probably cannot be taken at face value, as they most likely reflected data entry errors on the part of VMWs. Under the URC system (and as distinct from the FHI 360 system), VMWs were required to transport day-0 and day-3 slides together on day-3. The majority of slides (92%) were then either transported to the health centre on the same day or on the following day (i e, day-four). Once received, 86% of slides were examined at the health centre either on the day they were received or the day after. Only a small minority of slides (6%) had not been examined after two days. The longest delay between a slide being received and examined was six days.

To determine the completeness of reporting through the day-3 surveillance system, the total number of day-0 slides prepared at each site over the course of the pilot was compared to the total number of RDT-positive tests reported by the same VMWs through the routine national reporting system. For Pailin and Battambang the number of day-0 slides examined (n = 271) matched very closely the total number of positive RDT results reported by VMWs through the routine system (n = 308), indicating that a large proportion of *P. falciparum-*positive individuals were being effectively captured by the day-3 surveillance system. At Pramaoy, however, only 27 day-0 slides were examined over the course of the pilot, compared to 713 *P. falciparum* or mixed infections diagnosed by RDT by the same VMWs. This indicates that under the CNM system a very large number of *P. falciparum*-infected individuals were not being screened through the day-3 surveillance system.

### Experiences of implementation

As noted previously, individual day-3 surveillance pilots were carried out within a general framework that provided implementing partners with a significant amount of scope to adjust their protocols according to local contextual factors, as well as their own ideas about what would work best logistically. As a consequence, many key system characteristics (e g, procedures for patient follow-up and slide transport, staffing arrangements and use of financial incentives) varied considerably between partners and individual sites.

Within this study, semi-structured interviews were carried out with a representative cross-section of key informants, including 32 VMWs and 20 staff at health facilities. The purpose of the evaluation was to document the protocols adopted by each partner, to assess how these were interpreted and implemented on the ground and to gather feedback on the experience of implementing the day-3 system. While it is not within the scope of this paper to provide an exhaustive description of the evaluation findings, the following sections provide an overview of the principal variations between the partners’ protocols and a summary of the key areas of feedback received from staff involved with the implementation of these protocols.

### Remuneration of VMWs and health facility staff

The size and type of payments made to VMWs and health facility staff varied significantly between implementing partners. Under the FHI 360 and URC systems VMWs received payments for transporting day-0 and day-3 slides to their supervising health centre (between $2 and $8 per trip, depending on distance). Under the FHI 360 system VMWs were paid an additional $0.25 for each blood slide obtained but no payments were made to cover travel associated with patient follow-up (for DOT or the day-3 slide) or for communication. Under the URC system VMWs did not receive a per-slide payment but were provided with $4 per case to cover travel to provide DOT (i e, $2 per visit), plus $2 per month for communication. Within the CNM pilot system VMWs were entitled to a flat-rate payment of $5 per month to cover all day-3 surveillance activities and associated travel, although in practice this was not always received.

Financial arrangements also varied at supervising health centres. Under URC, laboratory staff (only) received a $5 monthly payment to cover communication costs but no payments were made for slide examination. Under FHI 360, supervising health centres were allocated $2 per month for communication and laboratory staff received $0.25 per blood slide examined, costings which were based on consultation with VMWs and health centre staff prior to the pilot. No direct payments were made to health centre staff under the CNM system.

None of the VMWs interviewed considered payments provided through the day-3 system to constitute an ‘incentive’ to carry out the extra tasks involved. At Ta Sanh and Pailin the prevailing view was that rates of travel-related compensation were realistic but did no more than cover actual costs incurred. Where extra payments were made for slide preparation (under FHI 360), these were universally seen as being insufficient compensation given the amount of work involved. Instead a large majority of the VMWs and health centre staff interviewed alluded to the importance of non-financial motivating factors, including increased knowledge, training and the opportunity to better serve their communities. Most VMWs felt that the main benefit of the pilot was the provision of better case management to people in their communities.

### Slide preparation and transport

All VMWs in pilot villages received initial training on blood slide preparation and were required to prepare smears for all patients testing positive for *P. falciparum* or mixed infections by RDT. The majority of VMWs interviewed in Pailin reported some previous experience in preparing slides but this was not the case for VMWs in Battambang and Pursat. By the end of the pilot all VMWs interviewed stated that they were willing to prepare blood slides but the majority felt that they needed additional training and/or practice. The consensus among laboratory staff was that although there were a number of problems with VMW smears at the beginning of the pilot (typically an inappropriate quantity of blood), slide quality improved over the course of the project. At Ta Sanh, where a system of refresher training linked to routine VMW meetings was introduced, the microscopist estimated that by the end of the pilot 80-90% of incoming slides could be rated as good quality. In Pailin and Trang, where *P. falciparum* case numbers were very low, VMWs and laboratory staff noted that it was difficult to develop and maintain skills because opportunities for practice were rare.

As noted above, a distinctive element of the FHI 360 pilot was that day-0 and day-3 slides were transported individually, on the day of preparation, to the VMWs’ supervising health centre. Under the URC and CNM systems VMWs were required to send day-0 and day-3 slides together (on day-3). In the event both the URC and FHI 360 systems appeared to facilitate timely transfer of blood slides (see Table 2) and, on the basis of slide examination results for URC, the practice of transporting day-0 and day-3 slides simultaneously seems to be viable. Although no relevant process data were available for the CNM pilot, interviews with VMWs indicated that many slides prepared at village level were either never transported to the relevant health centre or were only taken at the time of the routine monthly VMW meeting. Unlike the FHI 360 and URC systems, VMWs under the CNM pilot were not compensated directly for the transport of slides.

Many VMWs reported that obtaining day-3 slides was challenging, as many patients were not prepared to make an appointment to return to the VMW’s house. Within the URC and FHI 360 systems (under which compensation was predicated on the VMW obtaining a day-3 slide), all VMWs stated that they were willing to obtain follow-up slides at the patient’s house. Under the CNM system, where a flat rate of compensation was in place, none of the VMWs interviewed had attempted to follow the patients up at home and, as very few patients were willing to return to the VMW on day-3, the vast majority of *P. falciparum* cases were effectively lost to follow-up as a result.

### Treatment with DOT

Among VMWs the level of awareness concerning the importance of DOT and adherence to set treatment schedules was consistently high across all sites. However, there was considerable variability between (and sometimes within) pilot sites in terms of actual provision of DOT. At Ta Sanh, all VMWs reported carrying out DOT for all patients. This was mostly achieved through home visits, for which VMWs were compensated directly. In Pailin, where no compensation was linked to DOT, VMWs typically asked *P. falciparum* cases to return for treatment on day-one and day-two; the majority view was that this was only workable because most patients lived close by. VMWs in Pailin appeared willing to treat patients at home in a minority of cases, but felt this was only viable because cases numbers were very low. At Pramaoy none of the VMWs interviewed had attempted to provide DOT at the patient’s home and only one VMW had attempted to provide DOT through appointments at the VMW station.

A number of VMWs raised concerns about providing DOT to migrant workers either because of practical difficulties associated with tracing individuals or the relatively high risk of individuals leaving the area before completion of the treatment regimen. Several VMWs noted the need for general flexibility in determining when, and when not, to attempt DOT, based on the likelihood of successful follow up.

### Slide examination and case reporting

Process data for the FHI 360 and URC pilots (Table 2) indicate that across the two sites 85% of blood slides were examined either on the same day or the day after they were received by the health centre (corresponding data for the CNM pilot are not available). However, this statistic belies a number of problems reported by health centre staff. In Pailin, among the three health centres visited during the evaluation one did not have trained staff capable of examining slides and staff at another claimed to be too busy with other ongoing activities to examine slides from the day-3 pilot (in both cases slides were routinely transported to an alternative health centre which effectively took on all microscopy associated with the day-3 pilot). Several of the laboratory officers interviewed reported being demotivated by the fact that either no incentives (under URC and CNM), or very modest incentives (under FHI 360), were paid for examining blood slides and one health centre chief considered this demotivating effect to be a serious risk to the overall viability of the day-3 system. The majority of laboratory staff interviewed felt that competition from other roles (often associated with more generous incentives) to be a major constraint on their ability to support the day-3 pilot.

Within the pilot system laboratory staff were also primarily responsible for sending SMS alerts of day-3 positive cases using a pre-defined coding system. With the exception of staff at one health centre who were unable to send texts using a non-Khmer script, none of the individuals interviewed reported any difficulties composing or sending the SMS.

### Coordination, supervision and feedback

Arrangements for project coordination differed between sites. Pilot activities in Battambang and Pailin were coordinated by specific provincial level staff within URC and FHI 360, respectively. All VMWs at these sites claimed to be very satisfied with the support and supervision provided, although a substantial number complained about a lack of feedback of individual slide results and a need for more guidance on case management of day three-positive individuals. Within the CNM system overall coordination was provided by national programme staff based in Phnom Penh and no attempt was made to introduce a system for supervision at the provincial level. This was reflected in feedback from VMWs in Pramaoy, the majority of whom considered the level of supervision and support they had received to be inadequate.

## Discussion

day-3 surveillance pilot activities have provided valuable data on rates of day-3 *P. falciparum* positivity in key areas of western Cambodia and also on the programmatic viability of community-based systems for detecting and reporting day-3 positive cases. The overall rate of day-3 positivity across all pilot sites was 21%, with the largest number of both day-0 and day-3 positive cases coming from a single site (Ta Sanh). This figure greatly exceeds not only the 3-5% background rate of day-3 positivity that might be expected in the absence of resistance to artemisinin, but also the 3-10% range which in the past has been seen as appropriate window for initiating containment activities [5]. Data from these community-based pilots are consistent with those published from therapeutic efficacy studies [8] which suggest that in key areas of western Cambodia rates of day-3 positivity far exceed the threshold figure of 10% which has been suggested as a possible indicator for the presence of drug resistance [4]. While attention has been previously focused on Pailin, data from these pilots indicate high levels of day-3 positivity are also characteristic of parts of neighbouring Battambang.

Community case management of malaria through village volunteers has been identified as a key mechanism for achieving targets relating to prompt access of appropriate malaria treatment [9] and as a prerequisite for malaria elimination [10]. The viability of using community-based volunteers to prescribe appropriate treatment on the basis of RDT diagnosis has been demonstrated in a variety of settings [11-22] and, as of 2010, 42 malaria-endemic countries have reported some degree of implementation of RDTs at community level [9]. Cambodia has a particularly long history of community-based diagnosis and treatment of malaria using VMWs [7] and there is good evidence that this has been an effective mechanism for improving access to appropriate diagnosis and treatment [23]. In areas of potentially emergent drug resistance the detection and follow-up of individuals with prolonged parasite clearance times would seem to be a logical and justifiable extension of the role of VMWs; however findings from this evaluation have also highlighted the non-trivial nature of implementing and sustaining day-3 surveillance activities. Effective day-3 surveillance is a highly intensive activity that introduces new roles and responsibilities (as well as significant extra work load) to VMWs and health facility staff. For the system to be effective patients testing positive for *P. falciparum* on day-0 need to be successfully traced and re-tested on day-3 and adherence to the relevant ACT regimen needs to be assured through the administration of DOT. In any event, not all of these elements were achieved consistently at all of the pilot sites.

Results presented in the previous section focused on key variations in protocols and experiences among different arms of the pilot; however a consistent finding from interviews conducted at all sites was the very high level of project acceptability and near universal support for the public health rationale of the project. There was clearly an appetite on the part of VMWs and health centre staff to improve the quality of care provided for malaria cases and day-3 surveillance was seen to contribute directly to this through its emphasis on treatment and monitoring of parasite clearance. Notably, however, these high levels of acceptability did not always translate into high levels of engagement. For most VMWs and facility staff, issues relating to existing workloads, financial incentives and availability of basic supplies created practical limits to the amount of time and effort they could justify in supporting the project.

A clear outcome from the pilot phase has been a strong evidence base to support the feasibility of surveillance predicated on blood slides obtained by VMWs. VMWs at all sites were willing and able to prepare blood films as part of their routine activity and with appropriate training VMWs with no relevant experience were capable of producing good quality smears by the end of the pilot. The amount of training input needed to achieve this should not be underestimated, however, and regular refresher training and supervision are required, particularly in very low transmission settings where VMWs have limited opportunity to practice these skills. This is consistent with experience in Vietnam, where village health workers are routinely expected to prepare blood films but lack of appropriate training has been identified as a key determinant of poor performance [24]. It is worth noting that within the URC pilot all refresher training was carried out within the existing system of supervision and support via routine monthly meetings.

Evidence from the evaluation suggests that effective DOT is achievable but requires a high level of motivation on the part of VMWs. There was considerable variability between (and sometimes within) pilot sites in terms of VMW provision of DOT. At Ta Sanh, VMWs routinely provided DOT and travelled to patients’ houses to do so. At all other sites arrangements were more variable, but in practice VMWs often opted to make appointments for patients to return to the VMW station for treatment. Some VMWs reported using accompanying friends and relatives to administer DOT. Others clearly had never attempted to provide DOT. Overall, the evidence points to the fact that comprehensive provision DOT cannot be achieved unless VMWs are provided with the resources (finance and transport) to allow them to follow up patients at home. It is unrealistic to expect all patients to re-visit VMWs on the second and third day to receive treatment. Indeed, instituting such a system would arguably be counter-productive and increase rates of non-compliance. Any system of DOT would also have to retain some flexibility and autonomy on the part of VMWs as to when and where DOT is achievable. Because under these circumstances the universal use of DOT cannot be assured, the validity of VMW-reported day-3 positivity rates should also be interpreted with caution.

On the evidence of process data and user feedback there was substantial variation between the sites in terms of the basic operational effectiveness of the pilot system. The most obvious distinction was between pilot activities carried out under the auspices of the non-governmental organisations (URC and FHI 360) and those supervised directly by CNM. In many ways the CNM system represented a ‘minimum’ model of implementation with comparatively low rates of remuneration for staff involved and with relatively modest inputs in terms of supervision. It is likely that the relatively smooth running of pilot activities under URC and FHI 360 can be attributed, at least in part, to strong supervisory support provided at the provincial level. In contrast, some of the problems experienced at Pramaoy could be linked to the absence of equivalent support mechanisms below the central level. Linked to this, it is also likely that pilot experiences were influenced strongly by the underlying characteristics of the VMW networks at each site. Most notably at Pramaoy, where the pilot was particularly ineffective, a large majority of VMWs complained about frequent (and sometimes chronic) stock-outs of ACT and RDTs, as well as problems with the supply of consumables for the day-3 pilot itself, both of which were symptomatic of weak supervision. For community volunteers the demotivating effect of such factors linked to their working environment have been clearly demonstrated in other settings [11,25].

Given the intensive nature of day-3 surveillance, the effectiveness of the system is linked directly to the performance of VMWs and key health centre staff (particularly laboratory staff), which itself will reflect a variety of individual-level and contextual factors [26,27]. Health worker motivation has been identified as an important direct and indirect determinant of performance and is therefore considered to be a key area for intervention. Previous reviews have highlighted the importance of financial incentives; however these alone may not be sufficient to motivate health workers [28-30], particularly in the case of community volunteers who are by definition non-salaried. Within the current evaluation none of the VMWs or health centre staff interviewed considered the payments provided through the pilot system to constitute an incentive. A number of VMWs pointed out that financial considerations were not a primary factor in their original decision to volunteer and most were keen to highlight the satisfaction they derived from serving their community and/or to their increased level of knowledge through training. At the same time it is clear that realistic payments for the extra tasks are a prerequisite for the operational viability of VMW-centred initiatives such as this. Although VMWs felt that payments were too low to fully compensate for the time and effort involved, they were generally considered sufficient to cover their basic costs. Most VMWs made it clear that they would be unable to continue following up malaria cases in the absence of these basic payments. A number of VMWs and health centre staff also stressed the importance of payments being linked explicitly to specific activities such as slide transport or patient follow-up. Within the CNM pilot it was evident that use of a flat-rate monthly payment to VMWs was not conducive to increasing levels of VMW engagement with pilot activities. Moreover, key aspects of VMWs’ practices within the URC and FHI 360 pilots could be linked to the payment structures used in each case. Under FHI 360, for example, VMWs were not paid to travel to patients’ houses to administer DOT and as a result patients were required to travel to the VMW’s house to receive treatment. This proved to be viable in Pailin but would not be recommended as a general model of implementation. Under URC, on the other hand, VMWs routinely carried out active follow-up of patients and a number stated explicitly that this would only be possible while they received financial compensation for doing so. At both sites compensation was contingent on a day-3 slide being submitted to the supervising health centre which no doubt contributed to very high rates of day-3 follow-up (as distinct from experiences at the CNM site).

Typically when asked what was good about the project, or what motivated them to be involved, VMWs focused on improved case management. However, many felt they were not receiving sufficient support through training, guidance or supervision to maximize their effectiveness in this area. It has been shown in other settings that provision of adequate training and appropriate job-aids are critical to health worker performance [11,20,31] and there is a need to examine areas of best practice from these pilots and similar studies to tailor these elements in future. As noted above, adequate supervision is also a key factor influencing health worker performance. This fact is widely recognized, although supervision systems for community volunteers are nevertheless typically weak [27]. On a positive note, experience from the current pilots indicates that many of the training and supervision inputs required to support day-3 surveillance activities can be achieved through the existing mechanism of VMW monthly meetings. In addition, relatively small changes in the way health centre staff provide feedback to VMWs may produce beneficial effects. Perhaps somewhat counter-intuitively the amount of effort required to implement and sustain day-3 surveillance activities is likely to be greater in low transmission settings than in areas where *P. falciparum* cases are relatively common.

Within the piloted systems much of the burden of activity rests with VMWs but the viability of the system also depends on effective engagement by key health centre staff, and most notably laboratory staff responsible for blood slide examination. Within this evaluation feedback from laboratory staff interviewed was quite variable, particularly regarding the importance (or otherwise) of financial incentives. However, there was a clear general consensus that the degree to which laboratories could support day-3 surveillance was limited inherently by pre-existing commitments to other projects and routine activities, and in many cases staff were reluctant to take on extra duties. It is clear that any attempt to scale up new surveillance activities will need to address this constraint and, where required, strengthen laboratory capacity. The issue of competing commitments is also highly relevant to VMWs who in addition to their own personal commitments are now required to treat acute respiratory infections and cases of diarrhoea [6] and in some cases may also be responsible for the delivery of other services, including maternal and child health and family planning. It has been suggested that in the future community volunteers may represent a suitable platform for community-based approaches for reducing malaria transmission, including active case detection, focused screening and treatment and targeted focal vector control [10]. However, the capacity of VMWs to support such activities requires careful review prior to developing new policy in this area.

It is worth noting that in the early planning stages of the day-3 surveillance pilots, the novel use of SMS messaging to provide rapid alerts of day-3 *P. falciparum* cases to CNM and other stakeholders was a prominent feature of planned activities. In practice, however, this technological element represented a very small component of the overall work flow and any problems users had with the system (e g, inexperience in sending texts) appeared to be entirely tractable. It is important to recognize that within the current day-3 framework information technology (IT) is used essentially to increase the effectiveness of surveillance data once they have been generated. IT does not make the gathering of these data any easier. In reality the principal impediments to achieving effective surveillance of day-3 *P. falciparum* cases relate to basic health system constraints (outlined above) and there are clear limits to the extent that technology can support this process. In the elimination literature there has been a tendency to see the strengthening of surveillance systems as a technical challenge involving the deployment of new means of managing and transmitting surveillance data [32-34]. Gains from these developments will only be realised, however, once wider health system constraints have been effectively resolved.

On balance, evidence from this evaluation suggests that introducing community-based systems for detecting and reporting day-3 positive cases of *P. falciparum* is viable, but that a number of prerequisites need to be successfully met before such a system can be considered to be practically feasible. At the most basic level pre-existing local VMW-health centre networks need to be effective, incorporating adequate supervision and resupply of RDTs, drugs, slides and other consumables. Relatively frequent training of VMWs is required to ensure that slide quality is adequate and to fully empower VMWs in the process of case management. Given the relatively intensive nature of day-3 surveillance activities it is essential that VMWs are directly compensated for travel associated with obtaining and delivering blood slides and administering DOT. Careful consideration needs to be given to existing time commitments of both VMWs and health centre staff and where necessary human and operational capacity needs to be strengthened before such add-on activities can be supported. This latter point is pertinent not only in the case of day-3 surveillance but also in the wider context of using community volunteers to support interventions aimed at drug resistance containment and/or malaria elimination.

## Conclusions

Results from pilot activities indicate that an appropriately resourced and well-supported community-based system for day-3 surveillance is certainly capable of providing robust indicator data on day-3 positivity. However, on its own such a system cannot be considered comprehensive as it fails to capture *P. falciparum* cases presenting directly to government facilities or private providers. Creating a more comprehensive day-3 surveillance system would involve effective integration of community- and facility-based systems. A suitable blueprint for such a system is relatively easy to envisage. In practice, however, achieving the sort of close coordination of facility- and village-based activities required to make this work would be more challenging. The question of whether there is proper justification for this type of integrated system depends ultimately on the perceived purpose of day-3 surveillance. If it is primarily to serve as a platform for monitoring temporal changes in day-3 positivity rates then arguably an extensive sentinel site system, most likely consisting of inpatient facilities, would almost certainly be a more efficient and cost-effective approach to generating suitable datasets. If the purpose of day-3 surveillance is to identify as many *P. falciparum* infections in the community as possible, and use this information to clear these and nearby infections, other mechanisms (e.g*.* FSAT) may be more appropriate. If, however, the rationale for the day-3 system is to provide an effective alert system for identifying potential clusters of day-3 positives, the type of system piloted in this exercise will be potentially valuable, providing they are appropriately targeted geographically and linked explicitly to well-defined plans for response. Whatever option is selected for scale-up, this study underlines the importance of evaluation in assisting programmes and donors to make rational decisions.

## Abbreviations

ACT: Artemisinin-based combination therapy; ARCE: Strategy for the containment of artemisinin-tolerant malaria parasites in Southeast Asia; CNM: National centre for parasitology, entomology and malaria control; DOT: Directly observed therapy; IT: Information technology; RDT: Rapid diagnostic test; VMW: Village malaria workers.

## Competing interests

The authors have declared that they have no competing interests.

## Authors’ contributions

JC, LDS, KST, SN, and SM conceived and designed the study. JC, LDS, TB, KST, and SN participated in the implementation and coordination of the study. JC performed the data analysis. All authors contributed to the writing of the manuscript and have approved the final version.
